# Citation metrics and scientometrics

**DOI:** 10.17305/bb.2023.10233

**Published:** 2024-04-01

**Authors:** Rajko Igić

**Affiliations:** 1 Academy of Sciences and Arts of the Republic of Srpska, Banja Luka, Bosnia and Herzegovina; 2 Medical Center, Sombor, Serbia

I read the article “Scientometrics and academia” by Dr. Zerem and colleagues [1]. My perspective on citation metrics and scientometrics is more cautious. Therefore, in this article, I present my viewpoint on this subject.

Humans have always needed to count in their daily life. For this, they likely first used fingers and tally marks. The long-term use of numbers led to gradual improvements. The early numeral system, the base 60 or sexagesimal system, was developed by the Sumerians, adopted by the Babylonians, and is still used for measuring time, angles, and geographic coordinates. In the 12th century CE, the decimal or Hindu-Arabic number system was introduced to the West. Our tendency to count seems inherent; at times, it appears to border on arithmomania. With the advent of computing technology, counting became enormously easy, leading to new applications such as citation metrics or citatometry.

In 1955, Eugene Garfield conceived a new method for cataloging scientific literature, and in 1964, he established the Institute for Scientific Information (ISI) in Philadelphia [2]. This method of indexing disseminated scientific literature globally, and citatometry became the foundation for scientometrics, a quantitative study of science that evaluates publications and the scientific contributions of individual researchers, research groups, institutions, journals, and the science development in a country. Today, Clarivate Analytics continues to use this tool to spread scientific information.

Citatometry is based on the number of citations a work receives; more citations indicate a greater impact. Citation data can be obtained from specific databases and, more recently, from various alternative metrics like reviews, downloads, impact on health, and environmental benefits. However, questions about the reliability of using citation frequency as a measure of scientific progress persist. These concerns have been debated, and the limitations relate to the impact of a work or its quality [3]. For instance, biochemist Olive H. Lowry’s method for protein determination has been cited 305,148 times, far exceeding the total citations of any Nobel Prize winner [4]. In contrast, Albert Einstein’s works were cited 1,564 times during his lifetime (until 1955), and discoveries like those of Milutin Milanković received “delayed recognition” during his lifetime [5]. Conversely, a lower-quality article might be cited to correct problematic data or opinions. Among the 7,125 highly cited scientists (1 among 1,000), many “position papers” exist, where all listed authors (e.g., 50, 100, 1,000) receive the same number of citations as the article, but only a few authors contribute [6]. In such multi-author papers, the number of self-citations is proportional to the number of authors.

Such inconsistencies in citatometry should be addressed. One solution is to present citation metrics separately for publications and authors. The authors should share the citations of the paper. This adjusted citation number, iC, would lead to a fairer distribution and eliminate so-called highly cited scientists who benefit at the expense of others. This approach would greatly assist scientometrics [7].

Despite citatometry’s shortcomings, scientometrics should not be abandoned as it provides short-term predictability of scientific impact [8]. Caution is necessary when using only citatometry to evaluate research publications [9]. Alternative metrics may help. Caution is particularly warranted in research institutions, funding agencies, and academia [1]. Ideally, evaluating scientific impact would involve reading each publication and determining its significance. However, the Web of Science tracks over 9,000 journals, and there are many more “lesser” journals not included in this database. It is impractical to find enough evaluators to read over a million papers annually, unless, perhaps, one day, wise robots will do so. Until then, classic methods of estimation often take 30, 60, or more years to recognize the most significant innovations and contributions to progress.

Let me illustrate with an example of how classic research assessment begins. When Professor U.S. von Euler visited the University of Tuzla in 1982 [10], I presented our publications on ACE activity in various eye structures [11–14]. He remarked, “I am familiar with these studies. ACE is crucial to the renin-angiotensin system, essential for cardiovascular and renal functions. When blood pressure falls, the kidneys release renin, forming angiotensin II, one of the most potent vasoactive substances, causing vasoconstriction and a rise in blood pressure. ACE in eye tissues suggests local angiotensin II production, regulating blood flow, including in the retina. Therefore, using ACE inhibitors in millions of hypertensive patients could benefit various eye diseases. This is an important discovery.” In 1996, Van Haeringen noted, “Ophthalmic literature on the RAS began in 1977 with a study by Igić and colleagues on detecting ACE activity in retina homogenates,” and in 2016, Choudhary et al. published a comprehensive literature survey on the RAS in the eye, retrieving 180 publications since 1977 [15, 16]. This reflects high interest in the subject and supports von Euler’s opinion. However, our publications on ACE and renin in the eye, from 1975 to the present, have only garnered 135 citations, far fewer than the several thousand citations an assistant reviewer might receive for contributing to a “position paper”. This raises the question of how citatometry can assist in the final evaluation of this and other discoveries.

Science is a collective endeavor of researchers across various fields, aiming to form theories and concepts through experimentation and observation, revealing nature’s secrets. The exponential increase in scientific literature does not always proportionally offer new directions or stimulate knowledge expansion [17].

In conclusion, to evaluate the value of research publications, classic assessment methods are needed in addition to citations and alternative metrics. However, as this approach is subjective, the final evaluation often comes after several generations of scientists have made their contributions. It is uncertain if the development of artificial intelligence will provide a fast and competent additional evaluation of every publication.
